# Postpartum Lifestyle Behaviors among Women with Hypertensive Disorders of Pregnancy: Data from the HUNT Study

**DOI:** 10.3390/ijerph20217025

**Published:** 2023-11-06

**Authors:** Ingrid Hafskjold, Vegar Rangul, Hanne Ringvoll, Marit Kolberg, Eirin B. Haug, Rune Blomhoff, Hege Berg Henriksen, Julie Horn

**Affiliations:** 1 Department of Public Health and Nursing, Faculty of Medicine and Health Sciences, Norwegian University of Science and Technology (NTNU), 7491 Trondheim, Norway; 2 Faculty of Nursing and Health Sciences, Nord University, 7600 Levanger, Norway; 3 Levanger Hospital, Nord-Trøndelag Hospital Trust, 7600 Levanger, Norway; 4 Center for Oral Health Services and Research Mid-Norway (TkMidt), 7030 Trondheim, Norway; 5 K.G. Jebsen Center for Genetic Epidemiology, Department of Public Health and Nursing, Norwegian University of Science and Technology (NTNU), 7491 Trondheim, Norway; 6 Department of Nutrition, Institute of Basic Medical Sciences, University of Oslo, 0316 Oslo, Norway; 7 Department of Clinic Service, Division of Cancer Medicine, Oslo University Hospital, 0424 Oslo, Norway; 8 Department of Obstetrics and Gynecology, Levanger Hospital, Nord-Trøndelag Hospital Trust, 7600 Levanger, Norway

**Keywords:** cardiovascular disease, gestational hypertension, lifestyle behavior, postpartum, pre-eclampsia, prevention

## Abstract

Hypertensive disorders of pregnancy (HDP) are associated with an increased risk of cardiovascular disease later in life. Clinical guidelines for postpartum follow-up after HDP often recommend lifestyle counseling to reduce this risk. However, knowledge about lifestyle behaviors and perceptions among women with a history of HDP is limited. We linked data from the fourth survey of the population-based Trøndelag Health Study (HUNT4) with data from the Medical Birth Registry of Norway. The associations between HDP and postpartum lifestyle behaviors and perceptions were examined using multivariable logistic regression. In a secondary analysis, HUNT4 participants with a recent history of pre-eclampsia were compared with women with a recent history of pre-eclampsia participating in a postpartum pilot intervention study. Lifestyle behaviors and perceptions were self-reported and included diet (intake frequency of fruits, vegetables, meat, fish, and sugar-sweetened beverages), alcohol intake, physical activity, sleep, smoking, lifestyle satisfaction, and the importance of a healthy lifestyle. Among 7551 parous HUNT4 participants, 610 had a history of HDP. We found no differences in lifestyle behaviors between women with and without a history of HDP. However, women with HDP had higher odds of being unsatisfied with their lifestyle. Women with pre-eclampsia participating in a postpartum lifestyle intervention study tended to have a healthier lifestyle at baseline than women participating in HUNT4. Future studies should explore how lifestyle intervention programs could be adapted to the needs of women who have experienced HDP or other pregnancy complications that are associated with an increased risk of CVD.

## 1. Introduction

Preeclampsia (PE) and gestational hypertension (GH) are hypertensive disorders of pregnancy (HDP) that complicate 7–10% of all pregnancies in Norway [1]. Women with a history of HDP are at an approximately two-fold higher risk of developing cardiovascular disease (CVD) later in life compared with women with normotensive pregnancies [2]. HDP and CVD share common risk factors, including adverse levels of body mass index (BMI), blood pressure, glucose, and lipids [3,4]. Previous studies have suggested that the increased risk of CVD in women with a history of HDP is mainly explained by unfavorable levels of modifiable cardiovascular risk factors, primarily body weight and blood pressure [5,6]. Thus, pregnancy complications, including HDP, may raise awareness of the increased maternal risk for CVD and encourage early preventive efforts [7].

There is substantial evidence that a healthy diet, physical activity, weight loss, alcohol moderation, and smoking cessation can reduce the risk of CVD [8,9]. The postpartum period has been suggested as a “window of opportunity” for healthy lifestyle changes, with the potential to reduce cardiovascular risk in both mothers and their offspring [10].

Several intervention trials have been developed to improve cardiovascular health in women with a history of HDP via postpartum lifestyle modifications [11,12]. However, preventive healthcare requires a comprehensive understanding of lifestyle behaviors. So far, knowledge about lifestyle behaviors among women with a history of HDP is limited, especially from large population-based studies. Nor do we know if the lifestyle behaviors of women recruited to intervention studies are different from those of women with HDP in general and to what degree study participants are representative of the target population. Previous studies have claimed that non-participants in lifestyle intervention programs could be those who would have benefited the most from the interventions [13].

In the present study, we used data from the Trøndelag Health Study (HUNT) linked to the Medical Birth Registry of Norway (MBRN) to evaluate postpartum lifestyle behaviors (≤20 years since last birth) in women with and without a history of HDP. Furthermore, we compared the lifestyle behaviors of women with a history of PE participating in a postpartum lifestyle intervention study with the lifestyle behaviors of HUNT participants with a recent history of PE.

## 2. Materials and Methods

### 2.1. Study Population

The main study population consisted of women who participated in the fourth survey of the Trøndelag Health Study (HUNT4), a population-based study conducted between 2017 and 2019 in the northern part of Trøndelag county in Norway [14]. HUNT4 gathered comprehensive information on participants’ general health and lifestyle behaviors, including smoking, alcohol intake, sleeping patterns, physical activity, and broad information on dietary intake. Using the unique identification number of all Norwegians, we linked the information from HUNT to data from the MBRN. The MBRN is a mandatory registry and contains comprehensive information on pregnancy and birth complications, including data on all deliveries in Norway from 16 weeks of gestation [15].

**Figure 1:**
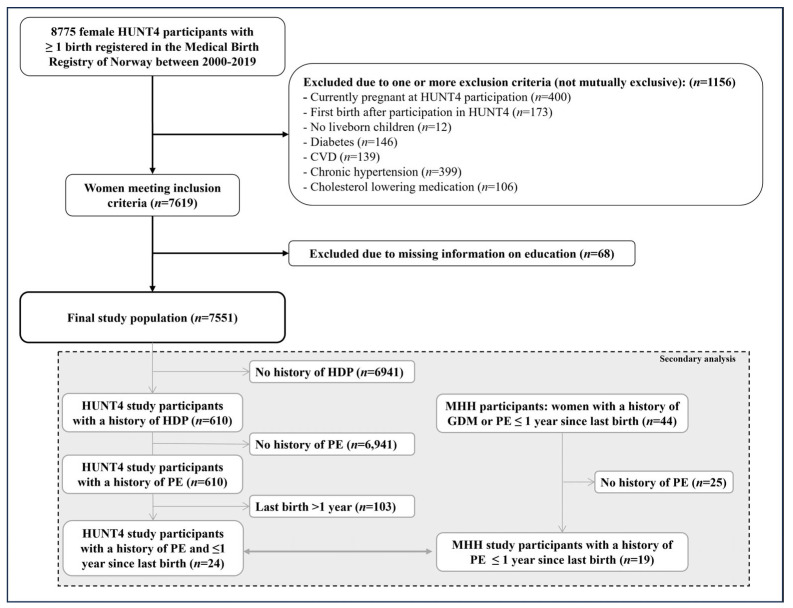
Flowchart of the study population. Abbreviations: CVD, cardiovascular disease; HDP, hypertensive disorders of pregnancy; HUNT4, 4th Trøndelag Health Study survey; PE, pre-eclampsia; MHH, Mom’s Healthy Heart study.

This study included 8775 women participating in HUNT4 who had at least one birth registered in the MBRN since 2000. We excluded 1156 women for the following, not mutually exclusive, criteria: Since we were interested in women’s postpartum lifestyles, we excluded women who were pregnant when participating in HUNT4 (*n* = 400) and women who gave birth to their first child after participating in HUNT4 (*n* = 173). We also excluded women with a history of CVD (*n* = 139), hypertension (*n* = 399), or diabetes (*n* = 146, self-reported via the HUNT questionnaire or registered in the MBRN), as well as women who reported the use of cholesterol-lowering medication (*n* = 106). We further excluded women without live-born infants (*n* = 12). Lastly, women with missing information on education were excluded (*n* = 68). The final study population consisted of 7551 women (Figure 1).

In a further step, we compared data on women participating in HUNT4 within one year after a pregnancy complicated by PE with baseline information on 19 participants of the Mom’s Healthy Heart study (MHH) with a history of PE. MHH is a pilot intervention study that has included women in Trøndelag with a prior diagnosis of PE or gestational diabetes within 3–12 months after giving birth from 2020 to 2021 [16]. All MHH study participants answered a baseline questionnaire including questions on meal patterns, alcohol consumption, smoking, sleep patterns, and physical activity. Out of the 610 women in HUNT4 with HDP, 449 women experienced PE, and 24 out of these gave birth within the last year before participating in HUNT4. These 24 women were compared with the 19 women in MHH who experienced PE (Figure 1).

### 2.2. Exposure and Covariates

Exposure was defined as a history of HDP (PE or GH in any pregnancy). In the MBRN, PE was defined as sustained de novo blood pressure elevation (systolic blood pressure of ≥140 mm Hg and/or diastolic blood pressure of ≥90 mm Hg) after 20 weeks of gestation along with proteinuria (≥0.3 g/24 h or a total protein/creatinine ratio of >0.3 or ≥1+ on a urine dipstick with a minimum of two measurements) [17]. GH was defined as transient hypertension during pregnancy or chronic hypertension identified in the latter half of pregnancy [17]. A validation study showed a positive predictive value of 90% for women registered with PE or GH in the MBRN between 1986 and 2012 [18].

Potential confounders were determined based on expert knowledge and directed acyclic graphs and included age at HUNT4 participation, parity (1 birth or ≥2 births), a self-reported family history of diabetes or CVD, highest obtained educational level (lower secondary, upper secondary, or tertiary), living with a partner or not, time since delivery (<1 year, 1–5 years, 5–10 years, and >10 years), and country of birth (Nordic country, other European country, or other).

### 2.3. Outcome

A range of lifestyle behaviors including diet, physical activity, smoking, and sleep quality were measured via questionnaires. All outcome variables were dichotomized following Norwegian national health guidelines as closely as possible to reflect adherence to healthy lifestyle recommendations [19,20,21]. The questions asked in HUNT and MHH were nearly identical for most variables except for diet. In MHH, a validated short food frequency questionnaire (NORDIET-FFQ) was used to assess dietary variables in both frequency and amount over the previous 1–2 months [22]. NORDIET-FFQ is a semiquantitative short 63-item food frequency questionnaire that was designed to measure adherence to the Norwegian food-based dietary guidelines. In HUNT4, participants only reported food frequencies. An elaborate overview of the definitions of the outcome variables is given in Table S1.

Physical activity was assessed based on information about the frequency, duration, and intensity of physical activity per week obtained from HUNT questionnaires or NORDIET-FFQ. Metabolic equivalents (MET) were calculated, and we divided them into two levels: above and below the international recommendations of at least 500 MET minutes per week, which reflects the amount of weekly physical activity necessary to achieve significant health benefits [20].

Participants reported their night-time sleep duration on a regular weekday, and a short sleep duration was defined as <6 h of sleep per night [21]. Women were also asked whether they felt difficulty coping during the daytime (socially or professionally) due to sleep problems (≥3 times per week or never/seldom).

Smoking was based on women’s current smoking status (yes/no).

Lifestyle perception was measured by asking “How important is it for you to live healthy (important/very important or less important/not important)?” and “How satisfied are you with your own lifestyle (diet, physical activity, smoking- and drinking habits) (very satisfied/satisfied or less satisfied/not satisfied)?”.

Questions about diet concerned how often fruit and berries, vegetables, red meat, fish, and beverages with added sugar (e.g., soda and squashes) were consumed.

Alcohol consumption was dichotomized into <7 or ≥7 units per week.

### 2.4. Statistical Analyses

The associations of HDP with lifestyle behaviors were examined using logistic regression. The results are presented as crude odds ratios with 95% confidence intervals (model 1). Multivariable analyses were adjusted for age at HUNT4 participation, parity, a family history of CVD, highest obtained education level, living with a partner or not, time since delivery, and country of birth (model 2).

The differences between the women participating in MHH and women participating in HUNT4 with a history of PE were tested using independent-sample t-tests for continuous variables or Fisher’s exact test for categorical variables. A *p*-value of ≤0.05 was considered statistically significant. The data analyses were performed in STATA 17.0 (StataCorp, College Station, TX, USA).

### 2.5. Sensitivity Analyses

In the sensitivity analyses, we separately examined postpartum lifestyle behaviors for women with a history of PE and women with a history of GH. To examine whether breastfeeding influenced our estimates, we performed a sensitivity analysis excluding women who were currently breastfeeding. Lastly, we restricted our analyses to women with <10 years since their last birth.

## 3. Results

### 3.1. Study Population

Table 1 presents the characteristics of the study population by HDP history. Among the 7551 study participants, 610 women (8%) were registered with a history of HDP in the MBRN. Compared with women with normotensive pregnancies, HUNT4 participants with a history of HDP were slightly older and had a higher BMI. Women with a history of HDP were also more likely to report a higher educational level and a family history of CVD.

### 3.2. Comparison of Lifestyle Behaviors According to History of HDP

The associations between a history of HDP and non-adherence to healthy lifestyle recommendations are presented in Table 2. Women with a history of HDP had lower odds of being current smokers (adjusted (adj.) OR 0.68 (95% CI 0.49–0.96)). We found no evidence of differences in diet or adherence to recommendations concerning physical activity between women with and without a history of HDP. Although not statistically significant, women with HDP tended to have lower odds of having a sleep duration of less than six hours (adj. OR 0.81 (95% CI 0.58–1.14)) and of having an alcohol intake of >7 units per week (adj. OR 0.35 (95% CI 0.11–1.10)). However, women with a history of HDP had higher odds of not being satisfied with their lifestyle (adj. OR 1.24; CI 1.03–1.48). Adjusting for BMI attenuated the estimate for not being satisfied with their own lifestyle to 0.96 (CI 0.79–1.16). Excluding women who were currently breastfeeding as well as restricting the analyses to women with <10 years since their last birth only slightly changed the estimates (Table S2). Analyzing separately for GH and PE only led to minor changes (Tables S3 and S4).

### 3.3. Comparison of Lifestyle Behaviors among Women with Recent PE According to Study Cohort

Table 3 presents the characteristics of the women with PE and ≤1 year since their last birth in the HUNT4 study and the women with PE participating in the MHH intervention study. Women participating in MHH were more likely to be primiparous (*p* < 0.001) and had a 0.7 kg/m^2^ higher BMI compared with HUNT participants with PE. MHH participants with PE had a higher proportion of highly educated women than the women in HUNT4 with PE.

Compared with MHH participants, HUNT4 participants were more likely to have an insufficient frequency of fruit intake (HUNT: 79.2% vs. MHH: 26.3%; *p* = 0.001) and vegetables (HUNT: 82.6% vs. MHH: 0%; *p* < 0.001). None of the women in HUNT4 but 42% of the MHH participants reported a sleep duration of <6 h per night (*p* = 0.001). Although not statistically significant, MHH participants tended to be less physically inactive (HUNT: 50.0% vs. MHH: 31.6%; *p* = 0.169) and more likely to rate a healthy lifestyle as less or not important (HUNT: 12.5% vs. MHH: 33.3%; *p* = 0.14) and more were not satisfied with their own lifestyle (HUNT: 37.5% vs. MHH: 57.9%; *p* = 0.23) than HUNT participants.

## 4. Discussion

Women with a history of HDP were less likely to be satisfied with their lifestyle, although they were less likely to be current smokers, and there were otherwise no significant differences in lifestyle behaviors between women with HDP and women with normotensive pregnancies.

There are sparse to no studies on postpartum lifestyle behaviors among women with a history of HDP. Timpka et al. conducted a cohort study in the United States, including 54,588 parous women from the Nurses’ Health Study II, to examine whether lifestyle risk factors modify the association between HDP and chronic hypertension [23]. In accordance with our results, the authors found no differences in physical activity and dietary patterns between women with and without a history of HDP. Our findings are also supported by a recent Norwegian study that reported no significant differences in physical activity levels one year postpartum between women with and without PE or GH [24]. However, previous studies on pre-pregnancy lifestyle and lifestyle early in pregnancy described an unhealthy diet and low physical activity level as risk factors for developing HDP [25,26]. One possible explanation is that women may have changed their lifestyle after a diagnosis of HDP. We had no data on pre-pregnancy lifestyle behaviors and were therefore not able to assess changes in lifestyle behavior. According to national guidelines for treatment and follow-up after HDP, a primary care follow-up program including lifestyle advice is recommended [17]. However, several previous studies have suggested that postpartum follow-up and counseling of women with HDP is suboptimal [27,28]. At the same time, a review study showed that these women are little informed about the diagnosis and its meaning for later risk of disease [28]. It is therefore unlikely that postpartum lifestyle modifications may explain the lack of differences in lifestyle behaviors in our study population. Our results, which showed that women with a history of HDP were at lower risk for current smoking, are in line with those from a meta-analysis in 2021, which suggested that smoking may be a protective factor for HDP [29].

Our finding that women with HDP reported lower lifestyle satisfaction than women with normotensive pregnancies was unexpected given the lower proportion of smokers among women with HDP and the lack of observed differences in other modifiable lifestyle behaviors between the two groups. This may indicate that there are smaller differences in lifestyle behaviors than our study was able to capture. Another possible explanation is the observed higher mean BMI in women with HDP as, especially in women, body image satisfaction and lifestyle satisfaction may be closely linked. This is supported by the attenuation of the estimate for lifestyle non-satisfaction after adjusting for BMI. Furthermore, women might be more aware of healthy lifestyle behaviors after experiencing HDP and therefore have a higher threshold for being satisfied with their own lifestyle.

We found that a high proportion of women with a history of PE in the HUNT population reported fruit and vegetable intakes below the recommended level. This is in accordance with data from a national survey indicating that most Norwegians should increase their intake of fruits and vegetables [30]. Compared with the HUNT study, MHH had a higher proportion of highly educated women, which has also been seen in other intervention studies [31]. We found that women with PE participating in the MHH intervention study were less likely to report insufficient consumption of fruits and vegetables than the HUNT4 participants with PE. A possible explanation for this could be that MHH recruited women with a healthier lifestyle. Previous lifestyle intervention studies targeted at different populations have repeatedly reported challenges in the recruitment of participants who have the most to gain or the most room for change [13,32]. However, the observed differences might also be explained by the different assessment methods of dietary patterns in HUNT and MHH.

The strengths of this study include the population-based design linked to the national birth registry. We were able to adjust our analyses for a wide range of potential confounders. Additionally, we were able to compare the lifestyle behaviors of women with a history of PE participating in a lifestyle intervention study with the lifestyle behaviors of women with PE from a population-based sample. However, this study has some limitations that should be addressed. First, the assessment of lifestyle behaviors was self-reported, and some misclassification is likely, especially for diet and physical activity. Future studies would benefit from objective measures of physical activity and more accurate dietary assessment methods, such as food frequency questionnaires. We were also unable to control for pre-pregnancy BMI and lifestyle behaviors as well as changes in these factors after pregnancy. Furthermore, variables were dichotomized due to small samples in subgroups; thus, we may have been unable to detect smaller differences in lifestyle behaviors. An additional limitation is that the questions on lifestyle perception may have been interpreted differently by the participants, leading to challenges in interpreting the results.

MHH assessed individuals’ dietary intakes with a short FFQ, including detailed questions concerning each food group and frequency as well as the amount, while HUNT used more general questions with fewer details solely focusing on frequency. This makes the two groups difficult to compare and affects the accuracy of the comparison, as it may have led to a difference in reporting fruit and vegetable intakes. The secondary analysis was limited by low power, and this, in turn, made it hard to perform an adjusted analysis.

In addition, given that lifestyle behaviors, and especially food consumption patterns, differ across countries [33,34], the findings in this Norwegian cohort may not be generalizable to other populations with different lifestyle behaviors. More research on postpartum lifestyle behaviors among women with HDP is needed in different populations to validate our findings.

## 5. Conclusions

Women with and without HDP had similar postpartum lifestyle behaviors. However, women with HDP were more likely to be unsatisfied with their lifestyle, possibly due to higher BMIs. There is still a need for more studies on the follow-up of women with a history of HDP.

Women included in the MHH intervention study, in general, had a healthier lifestyle than women with PE. This highlights the importance of targeted lifestyle intervention programs to reach women who have the most to gain. Our study provides new perspectives by approaching women who experienced HDP or other pregnancy complications that are associated with an increased risk of CVD. Future research is warranted to examine postpartum lifestyle behaviors in different populations and lifestyle behavioral changes from pre- to post-pregnancy.

## Figures and Tables

**Table 1:** Descriptive characteristics of parous HUNT4 study participants by history of HDP (*n* = 7551).

Characteristics		No History of HDP (*n* = 6941)	History of HDP (*n* = 610)
Age at participation		39.8 (7.8)	40.6 (7.2)
Ethnicity			
	Nordic	6477 (93.3%)	588 (96.4%)
	Other European	239 (3.4%)	13 (2.1%)
	Others	225 (3.2%)	9 (1.5%)
Education			
	Lower secondary education	181 (2.6%)	15 (2.5%)
	Upper secondary education	2400 (34.6%)	201 (32.9%)
	Tertiary education	4360 (62.8%)	394 (64.6%)
Household income			
	≤NOK 450,000	1079 (15.5%)	85 (13.9%)
	NOK 451,000–750,000	1686 (24.3%)	155 (25.4%)
	>NOK 750,000	2152 (31.0%)	196 (32.1%)
	Missing	2024 (29.2%)	174 (28.5%)
Living with partner		5908 (85.1%)	523 (85.7%)
Family history of CVD		1171 (16.9%)	122 (20.0%)
Family history of diabetes		1418 (20.4%)	145 (23.8%)
Parity			
	One birth	792 (11.4%)	63 (10.3%)
	≥Two births	6149 (88.6%)	547 (89.7%)
Time since last birth			
	<1 year	489 (7.1%)	39 (6.4%)
	1–5 years	1836 (26.4%)	149 (24.4%)
	5–10 years	1722 (24.8%)	156 (25.6%)
	>10 years	2894 (41.7%)	266 (43.6%)
Currently breastfeeding		423 (6.1%)	35 (5.7%)
BMI, kg/m^2^ (*n* = 7529)		26.1 (4.9)	28.0 (5.6)

The data are presented as means and standard deviation or n (%). Abbreviations: BMI, body mass index; CVD, cardiovascular disease; HDP, hypertensive disorders of pregnancy; HUNT4, 4th Trøndelag Health Study survey; NOK, Norwegian krone.

**Table 2:** Odds ratios for reporting non-adherence to healthy lifestyle recommendations and lifestyle perceptions among HUNT4 participants with a history of HDP compared with HUNT4 participants with normotensive pregnancies.

Lifestyle Behaviors		Model 1 *		Model 2 **	
		OR	95% CI	OR	95% CI
Lower adherence to diet recommendations					
	Fruit and berries (<7 times/week)	1.03	0.84–1.26	1.04	0.85–1.28
	Vegetables (<7 times/week)	1.07	0.89–1.29	1.09	0.90–1.32
	Red meat (>4 times/week)	1.00	0.78–1.29	1.06	0.82–1.36
	Fish (<1 times/week)	1.06	0.88–1.28	1.08	0.89–1.30
	Fatty fish (<1 times/week)	1.04	0.88–1.23	1.05	0.88–1.25
	Lean fish (<1 times/week)	1.04	0.88–1.23	1.04	0.88–1.23
	Alcohol (>7 units per week)	0.35	0.11–1.12	0.35	0.11–1.10
	Beverages with added sugar (>1 glass per week)	0.97	0.82–1.15	1.00	0.85–1.19
Physical activity (<500 MET minutes per week)		1.04	0.87–1.25	1.08	0.89–1.29
Sleep					
	Sleep duration (<6 h/day)	0.78	0.56–1.10	0.81	0.58–1.14
	Daytime dysfunction	0.91	0.59–1.39	0.93	0.60–1.42
Current smoker		0.69	0.50–0.96	0.68	0.49–0.96
Lifestyle perception					
	Healthy lifestyle less/not important	1.05	0.75–1.48	1.10	0.78–1.56
	Not satisfied with own lifestyle	1.19	0.99–1.40	1.24	1.03–1.48

* Model 1 is unadjusted. ** Model 2 is adjusted for age, ethnicity, highest obtained education, living situation, time since delivery, and parity. Abbreviations: CI, confidence interval; HDP, hypertensive disorders of pregnancy; HUNT4, 4th Trøndelag Health Study survey; MET, metabolic equivalent of task; OR, odds ratio.

**Table 3:** Sociodemographic, pregnancy, and lifestyle characteristics of study participants with a history of pre-eclampsia (HUNT4 vs. MHH).

Characteristics *		Women in HUNT4 with PE (*n* = 24)	Women in MHH with PE (*n* = 19)	*p*-Value
Age at participation		31.7 (4.6)	30.8 (4.4)	0.54
Country of birth				0.19
	Nordic	24 (100.0%)	17 (89.5%)	
	Other European	0 (0%)	2 (10.5%)	
Education				0.17
	Lower secondary education	0 (0.0%)	1 (5.3%)	
	Upper secondary education	9 (37.5%)	3 (15.8%)	
	Tertiary education	15 (62.5%)	15 (78.9%)	
Household income				0.53
	<NOK 450,000	4 (16.7%)	2 (10.5%)	
	NOK 451,000–750,000	5 (20.8%)	4 (21.1%)	
	>NOK 750,000	9 (37.5%)	13 (68.4%)	
	Missing	6 (25%)		
Living with partner		24 (100.0%)	17 (89.5%)	0.19
Family history of CVD		2 (8.3%)	13 (15.8)	0.64
Family history of diabetes		4 (16.7%)	4 (21.1%)	1.00
Parity				<0.001
	One birth	1 (4.2%)	12 (63.2%)	
	≥Two births	23 (95.8%)	7 (36.8%)	
Time since last birth (months)		6.6 (3.0)	8.2 (3.3)	0.10
Currently breastfeeding		20 (83.3%)	12 (63.2%)	0.17
Body mass index (kg/m^2^)		28.4 (5.8)	29.1 (5.9)	0.71
Diet				
	Fruit (<7 times/week)	19 (79.2%)	5 (26.3%)	0.001
	Vegetables (<7 times/week)	19 (82.6%)	0 (0%)	<0.001
	Red meat (>4 times/week)	4 (16.7%)	1 (5.3%)	0.36
	Fatty fish (<1 time/week)	12 (50.0%)	4 (21.1%)	0.06
	Lean fish (<1 time/week)	13 (54.2%)	7 (36.8%)	0.36
	Total fish (<1 time/week)	9 (37.5%)	2 (10.5%)	0.08
	Beverages with added sugar (>1 glass/week)	11 (47.8%)	12 (63.2%)	0.37
	Alcohol (>7 units/week)	0 (0%)	2 (10.5%)	0.21
Sleep				
	Sleep duration (<6 h/day)	0 (0%)	8 (42.1%)	0.001
Physical activity (<500 MET minutes/week)		9 (50.0%)	7 (36.8%)	0.52
Current smoker		0 (0%)	0 (0%)	-
Lifestyle satisfaction				
	Healthy lifestyle less/not important	3 (12.5%)	6 (33.3%)	0.14
	Not satisfied with own lifestyle	9 (37.5%)	11 (57.9%)	0.23

* The data are presented as means and standard deviation or n (%). Abbreviations: CVD, cardiovascular disease; HUNT4, 4th Trøndelag Health Study survey; PE, pre-eclampsia; MET, metabolic equivalent of task; MHH, Mom’s Healthy Heart study; NOK, Norwegian krone.

## Data Availability

Due to restrictions imposed by the HUNT Research Centre (in accordance with the Norwegian Data Inspectorate), the data cannot be made publicly available. The data are currently stored in the HUNT databank, and there are restrictions in place for the handling of HUNT data files. The data used from the HUNT Study in research projects will be made available upon request to the HUNT Data Access Committee (kontakt@hunt.ntnu.no). The HUNT data access information (available here: http://www.ntnu.edu/hunt/data (accessed on 1 September 2023)) describes in detail the policy regarding data availability.

## Supplementary Materials

The following supporting information can be downloaded at https://www.mdpi.com/article/10.3390/ijerph20217025/s1: Table S1: Outcome variable categorization and basis of definition; Table S2: Odds ratios for reporting non-adherence to healthy lifestyle recommendations and lifestyle perceptions among HUNT4 participants with HDP who gave birth within the last 10 years (*n* = 343) compared with HUNT4 participants with normotensive pregnancies (*n* = 4034); Table S3: Odds ratios for reporting non-adherence to healthy lifestyle recommendations and lifestyle perceptions among HUNT4 participants with a history of GH only (*n* = 160) compared with HUNT4 participants with normotensive pregnancies (*n*= 6922); Table S4: Odds ratios for reporting non-adherence to healthy lifestyle recommendations and lifestyle perceptions among HUNT4 participants with a history of PE (*n* = 445) compared with HUNT4 participants with normotensive pregnancies (*n* = 7064).
