# Supplementary material for: Postpartum Lifestyle Behaviors among Women with Hypertensive Disorders of Pregnancy: Data from the HUNT Study
Source: Int J Environ Res Public Health. 2023 Nov 6;20(21):7025. doi: 10.3390/ijerph20217025 (PMC10648747; doi:10.3390/ijerph20217025)
Supplement: Supplementary file 1 [file ijerph-20-07025-s001.zip › ijerph-2640641-supplementary.pdf]

SUPPLEMENTARY MATERIALS

**Table S1. Outcome variable categorization and basis of definition**			
**VARIABLE NAME**	**HUNT4 questionnaire**	**MHH questionnaire**	**BASIS OF CATEGORIZATION**<br>(based of Norwegian food-based dietary guidelines (NFBDG))
**Fruit and berries**	How often do you normally eat fruit and berries *?<br>a) Less than 7 times per week.<br>b) $\geq$ 7 times a week<br><br>*amount unknown	How many times per week and how much did you eat of the following? *Big fruit, medium fruit, small fruit and berries.*	*Recommended in NFBDG:*<br>250g fruit and/or berries every day [1].<br>a) Frequency not compliant with recommendations<br>b) Frequency indicate compliance with recommendations
**Vegetables**	How often do you normally eat vegetables*?<br>a) Less than 7 times per week.<br>b) $\geq$ 7 times a week<br><br>*amount unknown	How many times per week and how much did you eat of the following? *Garlic, onion, tomato, tomato sauce, mixed salad and other vegetables.*	*Recommended in NFBDG:*<br>250g vegetables every day [1].<br>a) Frequency not compliant with recommendations<br>b) Frequency indicate compliance with recommendations (assume daily intake).
**Red meat**<br>(beef, pork, lamb, game)	How often do you normally eat red meat*<br>a) 3 times or less per week.<br>b) 4 times or more per week<br><br>*amount unknown	How many times per week and how much did you eat of the following? *Clean red meat and processed red meat.*	*Recommended in NFBDG:*<br>500g per week à 2-3 dinners and some on bread.<br>a) Frequency indicate compliance with recommendations.<br>b) Frequency not compliant with recommendations.

**Fish**<br>*Lean fish:*<br>(e.g. cod, pollock)<br><br>*Fatty fish:*<br>(e.g. salmon, trout, herring, mackerel, haddock on bread or for dinner)	How often do you normally eat lean fish*:<br>a) Less than once per week.<br>b) 1 or more times per week<br><br>How often do you normally eat fatty fish*:<br>a) Less than once per week.<br>b) 1 or more times per week<br>*amount unknown	How many times per week and how much did you eat of the following? *Fat fish, lean fish and processed fish.*	*Recommended in NFBDG:*<br>Fish for dinner 2-3 times per week. Approximately 300-450 grams fish per week. At least 200g should be fatty fish, as salmon [1].<br><br>a) Frequency not compliant with recommendations.<br>*b)* Frequency indicate compliance with recommendations.
Total fish	How often do you normally eat fish in general*?<br>a) Less than once per week.<br>b) 1 or more times per week<br>*amount unknown		a) Frequency not compliant with recommendations.<br>b) Frequency on either lean- or fatty fish intake indicate compliance with recommendations.
**Alcohol**	How often one drinks alcoholic beverages (beer, wine and liquor) per week.<br><br>a) < 7 units per week.<br>b) > 7 units per week.	How many times per week and how much did you drink of the following? *Beer and wine with alcohol, and liquor.*	*Recommended in NFBDG and Nordic guidelines:*<br>Intake should not exceed 10g (1 unit per day) for women and 20g (2 units per day) for men alcohol [1]. 1 unit is defined as 12 g alcohol corresponding to the alcohol content in one bottle of beer (330mL), one glass of wine (120mL), or one drink with liquor (40mL). The definition of a unit varies in different countries from approximately 8 g to 12 g.
**Beverages with added sugar (Soda etc.)**	How many glasses do you usually drink of beverages with added sugar?<br>a) Never/Rare.<br>b) 1 or more glasses per week	How many times per week and how much did you drink of the following? *Juice and other beverages with added sugar.*	*Recommended in NFBDG:*<br>Limit the intake of beverages and foods with added sugar to less than 10% of total energy intake [1].

**Physical activity**<br>***Moderate intensity***<br>(Fast gait, hard housework or other activity which makes one short of breath)<br>***High intensity***<br>(Jogging, cross-country skiing or other activities that makes one very short of breath)	How hard do you exercise?<br>(average) How many times per week do you exercise?<br>For how long do you exercise each time?	How many times per week were you physical active at moderate intensity and for how long? How many times per week were you physical active at high intensity and for how long?	The metabolic equivalent of task (MET) is the objective measure of the ratio of the rate at which a person expends energy, relative to the mass of that person. Moderate intensity gives at least 3 MET per minute and high intensity at least 6 MET per minute. Weekly MET was calculated by combining weekly MET of moderate and high intensity exercise: Mod. intensity: Times per week * minutes per time * 3 High intensity: Times per week * minutes per time * 6 *Recommended is approximately > 500 MET per week, but > 900 MET is favorable [2].*<br>a) < 500 MET per week.<br>b) > 500 MET per week.
**Sleep amount**	a) Not enough sleep (6 hours or less).<br>b) Enough sleep, > 6 hours per night.		*Recommended in guidelines and similar Norwegian research:*<br>> 6 hours of sleep per night is considered healthy, but 8-9 hours is preferably [3].
**Sleep affecting daytime function**	How often do you function poorly during day (socially/at work) due to insomnia?<br>a) Less than 3 times per week<br>b) b) 3 or more times per week.	How do you perceive your sleep quality lately?<br>c) Very good/median<br>d) Bad/very bad	
**Lifestyle importance**	How important is it for you to live healthy?<br>a) Very important, important<br>b) Less important, not important	How important is it for you to live healthy?<br>a) Very important, important<br>b) Less important, not important	

**Lifestyle satisfaction**	How satisfied are you with your own lifestyle (diet, physical activity, smoking- and drinking habits)?<br>a) Very satisfied, satisfied<br>b) Less satisfied, not satisfied	How satisfied are you with your own lifestyle (diet, physical activity, smoking- and drinking habits)?<br>a) Very satisfied, satisfied<br>b) Less satisfied, not satisfied	

1. Nordic Nutrition Recommendations. Available online: https://www.norden.org/en/publication/nordic-nutrition-recommendations-2012 (accessed on 14.09.2022).
2. Kaminsky, L.A.; Montoye, A.H. Physical activity and health: what is the best dose? *J Am Heart Assoc* **2014**, *3*, e001430, doi:10.1161/JAHA.114.001430.
3. Johnsen, M.T.; Wynn, R.; Bratlid, T. Optimal sleep duration in the subarctic with respect to obesity risk is 8–9 hours. *PloS one* **2013**, *8*, e56756, doi:10.1371/journal.pone.0056756.

Table S2. Odds ratios for reporting non-adherence to healthy lifestyle recommendations and lifestyle perception among HUNT4 participants with HDP and ≤10 years since last birth (n=343) compared to HUNT4 participants with normotensive pregnancies (n=4034)

Lifestyle behaviors	Model 1		Model 2	
	OR	95% CI	OR	95% CI
Non-Adherence to dietary recommendations				
Fruit and berries (< 7 times/week)	1.01	0.13-1.31	1.03	0.79-1.35
Vegetables (< 7 times/week)	1.08	0.84-1.38	1.11	0.86-1.43
Red meat (≥ 4 times/week)	1.17	0.86-1.59	1.28	0.94-1.75
Fish (< 1 times/week)	0.96	0.75-1.24	1.00	0.78-1.30
Fatty fish (< 1 times/week)	0.89	0.70-1.12	0.90	0.72-1.14
Lean fish (< 1 times/week)	0.97	0.78-1.21	0.98	0.78-1.22
Alcohol (> 7 units per week)	0.62	0.15-2.61	0.58	0.14-2.43
Beverages with added sugar (≥ 1 glass per week).	0.95	0.76-1.18	0.99	0.79-1.23
Physical activity (< 500 MET per week)	1.01	0.79-1.29	1.05	0.82-1.34
Sleep				
Sleep duration (< 6 hours/day)	0.70	0.44-1.12	0.74	0.46-1.19
Daytime dysfunction	0.72	0.39-1.34	0.74	0.39-1.38
Current smoker	0.55	0.33-0.92	1.03	0.79-1.35
Lifestyle perception				
Healthy lifestyle not important	1.11	0.73-1.70	1.20	0.78-1.86
Not satisfied with own lifestyle	1.07	1.85-1.35	1.10	0.87-1.39

Model 1 is unadjusted. Model 2 is adjusted for age, ethnicity, highest obtained education, living situation, time since delivery and parity.

Abbreviations: CI, confidence interval; HDP, hypertensive disorders of pregnancy; MET, Metabolic equivalent of task; HUNT4, 4th Trøndelag Health Study survey; OR, odds ratio

Table S3. Odds ratios for reporting non-adherence to healthy lifestyle recommendations and lifestyle perception among HUNT4 participants with a history of GH only (n=160) compared to HUNT4 participants with normotensive pregnancies (n= 6922)

Lifestyle behaviors	Model 1		Model 2	
	OR	95% CI	OR	95% CI
Non-Adherence to dietary recommendations				
Fruit and berries (< 7 times/week)	0.84	0.58-1.19	0.90	0.63-1.30
Vegetables (< 7 times/week)	0.89	0.63-1.26	0.97	0.69-1.37
Red meat (≥ 4 times/week)	0.72	0.42-1.24	0.78	0.46-1.34
Fish (< 1 times/week)	0.97	0.68-1.40	1.07	0.75-1.54
Fat fish (< 1 times/week)	0.90	0.65-1.26	0.95	0.68-1.32
Lean fish (< 1 times/week)	0.94	0.69-1.29	1.01	0.73-1.39
Alcohol (> 7 units per week)	omitted	-	-	-
Beverages with added sugar (≥ 1 glass per week)	0.77	0.56-1.06	0.79	0.57-1.09
Physical activity (< 500 MET per week)	0.91	0.64-1.29	0.90	0.63-1.27
Sleep				
Sleep duration (< 6 hours/day)	0.68	0.34-1.34	0.73	0.37-1.44
Daytime dysfunction	1.01	0.47-2.17	1.13	0.51-2.40
Current smoker	0.59	0.30-1.15	0.90	0.63-1.30
Lifestyle perception				
Healthy lifestyle not important	0.93	0.47-1.84	1.06	0.53-2.11
Not satisfied with own lifestyle	1.06	0.75-1.49	1.07	0.75-1.51

Model 1 is unadjusted. Model 2 is adjusted for age, ethnicity, highest obtained education, living situation, time since delivery and parity.

Abbreviations: CI, confidence interval; GH, gestational hypertension; HUNT4, 4th Trøndelag Health Study survey; MET, Metabolic equivalent of task; OR, odds ratio

Table S4. Odds ratios for reporting non-adherence to healthy lifestyle recommendations and lifestyle perception among HUNT4 participants with a history of PE (n=445) compared to HUNT4 participants with normotensive pregnancies (n=7064)

Lifestyle behaviors	Model 1		Model 2	
	OR	95% CI	OR	95% CI
Non-Adherence to diet recommendations				
Fruit and berries (< 7 times/week)	1.12	0.88-1.41	1.10	0.87-1.39
Vegetables (< 7 times/week)	1.15	0.92-1.43	1.14	0.91-1.42
Red meat (≥ 4 timess/week)	1.11	0.84-1.47	1.16	0.88-1.54
Fish (< 1 times/week)	1.09	0.88-1.35	1.08	0.87-1.34
Fatty fish (< 1 times/week)	1.09	0.90-1.33	1.09	0.89-1.32
Lean fish (< 1 times/week)	1.08	0.89-1.30	1.05	0.86-1.27
Alcohol (> 7 units per week)	0.49	0.15-1.55	0.47	0.15-1.48
Beverages with added sugar (≥ 1 glass per week)	1.06	0.88-1.28	1.09	0.90-1.32
Physical activity (< 500 MET per week)	1.09	0.89-1.35	1.15	0.93-1.42
Sleep				
Sleep duration (< 6 hours/day)	0.82	0.56-1.20	0.84	0.57-1.23
Daytime dysfunction	0.87	0.53-1.44	0.86	0.52-1.42
Current smoker	0.73	0.50-1.06	1.10	0.87-1.39
Lifestyle perception				
Healthy lifestyle not important	1.10	0.74-1.62	1.12	0.75-1.66
Not satisfied with own lifestyle	1.24	1.01-1.52	1.31	1.06-1.61

Model 1 is unadjusted. Model 2 is adjusted for age, ethnicity, highest obtained education, living situation, time since delivery and parity.

Abbreviations: CI, confidence interval; PE, preeclampsia; HUNT4, 4th Trøndelag Health Study survey; MET, Metabolic equivalent of task; OR, odds ratio
